# A call for better understanding the role of albumin in mediating VE-cadherin phosphorylation and endothelial barrier dysfunction in septic patients

**DOI:** 10.1186/s13054-021-03637-y

**Published:** 2021-07-01

**Authors:** Yupei Li, Luojia Jiang, Tao Song, Yiran Wang, Baihai Su

**Affiliations:** 1 Department of Nephrology, Med-X Center for Manufacturing, West China Hospital, Sichuan University, Guoxue Alley No. 37, Chengdu, 610041 Sichuan Province China; 2 Institute for Disaster Management and Reconstruction, Sichuan University, Chengdu, 610207 Sichuan Province China; 3 College of Polymer Science and Engineering, Sichuan University, Chengdu, 610065 Sichuan Province China; 4 The First People’s Hospital of Shuangliu District, Chengdu, 610200 Sichuan Province China

**Letter to editor** regarding “Endothelial damage in septic shock patients as evidenced by circulating syndecan-1, sphingosine-1-phosphate and soluble VE-cadherin: a substudy of ALBIOS” by Piotti et al. [1].

We read with great interest the article by Piotti et al. which showed that soluble VE-cadherin was independently associated with the need for renal replacement therapy during ICU stay and albumin supplementation lowered circulating VE-cadherin consistently over time, which might be partially attributed to an increase in intravascular volume as evidenced by higher NT-proBNP in septic patients receiving albumin supplementation [1]. While the authors briefly proposed the potential mechanisms underlying the endothelial protective effect of albumin (namely their anti-inflammatory and antioxidant properties), here we intend to add more discussion to fuel the understanding of the role of albumin in mediating VE-cadherin phosphorylation and endothelial barrier dysfunction in septic patients.

Cell–cell adherens junctions are regarded as the primary junctions in the peripheral microvasculature. VE-cadherin, the main component of adherens junction, is anchored to the actin cytoskeleton through catenins (α-, β-, γ-, and p120-catenin) and participate in the regulation of endothelial barrier integrity and permeability [2]. In sepsis, inflammatory mediators (namely lipopolysaccharide, cytokines, thrombin, and complement 5a etc.) could not only down-regulate the expression of VE-cadherin on the membrane of endothelial cells, but also impair the cytoskeleton-junction response characterized by myosin light chain phosphorylation and tyrosine phosphorylation of VE-cadherin through the activation of Src family kinases, which significantly contributes to adherens junction dissociation [3]. Meegan and colleagues further identified citrullinated histone 3 as a functional contributor to cell–cell adherens junction opening and cytoskeleton reorganization, causing microvascular endothelial barrier dysfunction [4]. Since a previous publication has suggested the remarkable histone-neutralization effect of albumin characterized by decreased histone-induced endothelial cell damage [5], we speculate that albumin might also alleviate the impaired cell–cell adherens junctions and endothelial barrier function possibly by antagonizing histone-mediated adherens junction loss and cytoskeleton reorganization. Of note, this protective effect of albumin might be independent of the expression of VE-cadherin on the surface of endothelial cells. Future explorations are thus encouraged to clarify the mechanisms underlying the effect of albumin supplementation in mediating tyrosine phosphorylation of VE-cadherin, preventing endothelial barrier dysfunction and improving survival in patients with sepsis.

## Data Availability

Not applicable.
