# Supplementary material for: A call for better understanding the role of albumin in mediating VE-cadherin phosphorylation and endothelial barrier dysfunction in septic patients
Source: Crit Care. 2021 Jul 1;25:232. doi: 10.1186/s13054-021-03637-y (PMC8252191; doi:10.1186/s13054-021-03637-y)
Supplement: Related texts

# Reply: Authors’ response

**Authors:** Pietro Caironi, Jennifer Marie Theresia Anna Meessen, Arianna Piotti, Roberto Latini

**Affiliations:** 5 grid.7605.4 0000 0001 2336 6580 Department of Anesthesia and Critical Care, AOU S. Luigi Gonzaga, Orbassano and Department of Oncology, University of Turin, Turin, Italy; 6 Department of Cardiovascular Medicine, Institute for Pharmacological Research Mario Negri IRCCS, Milan, Italy; 7 grid.4527.4 0000000106678902 Department of Biochemistry and Molecular Pharmacology, Mario Negri Institute for Pharmacological Research IRCCS, Milan, Italy

To the editor

We read with interest the letter by Yupei Li and colleagues, discussing a potential novel mechanism by which albumin might counteract the altered endothelial permeability characterizing patients with sepsis. In support to the histone-neutralizing effect of albumin, in cultured endothelial cells, we reported that higher PTX3 at day 1 was associated with greater hemodynamic instability, suggesting increase in PTX3 secretion as a compensatory response. Similarly, in patients receiving albumin supplementation, PTX3 appeared significantly lower than in patients receiving crystalloids [6]. In another in-vitro study, Otero K and colleagues reported that advanced-glycation end-product-modified bovine serum albumin (BSA), an abnormal form of albumin, induces endothelial dysfunction and increased permeability through VE-cadherin complex disruption, as observed in diabetes or aging, an effect not observed with normal BSA [7]. Taken together, these findings suggest that albumin, when normally functioning, may preserve endothelial permeability also by maintaining the integrity of VE-cadherin complex.

Despite these interesting speculations, the overall picture of the role of albumin in relation to endothelial permeability is likely more complex. Very often, hypoalbuminemia, during septic shock, is considered per se a proof of increased endothelial permeability, although this statement remains unproven. Indeed, during sepsis, hypoalbuminemia is a common finding often associated with an increased risk of death, but several data have shown that during systemic inflammation albumin transcapillary escape rate may not be different from that of healthy subjects [8]. Moreover, even the association between glycocalyx disruption, as denoted by elevated levels of syndecan-1, and endothelial permeability has been challenged [9]. Finally, volume expansion with 20% albumin as observed in clinical conditions characterized by increased endothelial permeability, such as in patients with burns, appeared similar to that observed in normal conditions [10].

Independent of the mechanisms potentially involved, albumin supplementation targeting the restoration of its normal serum concentration appears a reasonable approach during sepsis. When compared to a fluid strategy including only crystalloids, in the ALBIOS trial, albumin replacement appeared associated with a faster normalization of cardiovascular dysfunction, as well as with a reduced net positive fluid balance during the resuscitation phase. Whether or not these advantages are related to ancillary functions of albumin (in addition to its primary oncotic property) remains unproven. We are currently conducting a novel multicenter randomized controlled trial, the ALBumin Italian Outcome Septic Shock—BALANCED (ALBIOSS-BALANCED) trial (#NCT03654001), comparing, in patients with septic shock, albumin replacement in addition to crystalloids to the use of only crystalloids over the first 90 days of treatment. Already 400 patients have been enrolled and we do hope that the results of such a trial, together with the ARISS trial (Albumin Replacement Therapy in Septic Shock, #NCT03869385) ongoing in Germany, will definitely prove whether albumin supplementation in septic shock improves survival.
